# Incidence and outcome of myocardial infarction treated with percutaneous coronary intervention during COVID-19 pandemic

**DOI:** 10.1136/heartjnl-2020-317685

**Published:** 2020-10-06

**Authors:** Moman A Mohammad, Sasha Koul, Göran K Olivecrona, Matthias Gӧtberg, Patrik Tydén, Erik Rydberg, Fredrik Scherstén, Joakim Alfredsson, Peter Vasko, Elmir Omerovic, Oskar Angerås, Ole Fröbert, Fredrik Calais, Sebastian Völz, Anders Ulvenstam, Dimitrios Venetsanos, Troels Yndigegn, Jonas Oldgren, Giovanna Sarno, Per Grimfjärd, Jonas Persson, Nils Witt, Ellen Ostenfeld, Bertil Lindahl, Stefan K James, David Erlinge

**Affiliations:** 1 Department of Cardiology, Clinical Sciences, Lund University, Skåne University Hospital, Lund, Sweden; 2 Department of Cardiology, Linköping University, Linköping, Sweden; 3 Department of Medicine, Växjö Hospital, Växjö, Sweden; 4 Department of Cardiology, Department of Molecular and Clinical Medicine, Department of Cardiology, Sahlgrenska University Hospital, Institute of Medicine, Department of Molecular and Clinical Medicine, Sahlgrenska Academy at University of Gothenburg, Gothenburg, Sweden; 5 Department of Cardiology, Faculty of Health, Örebro University, Örebro, Sweden; 6 Department of Cardiology, Östersund Hospital, Östersund, Sweden; 7 Department of Cardiology, Karolinska University Hospital, Stockholm, Sweden; 8 Department of Medical Sciences and Uppsala Clinical Research Center, Uppsala University, Uppsala, Sweden; 9 Department of Internal Medicine, Västmanlands Sjuk, Lund, Sweden; 10 Division of Cardiovascular Medicine, Department of Clinical Sciences, Karolinska Institutet, Danderyd University Hospital, Stockolm, Sweden; 11 Dvision of Cardiology, Department of Clinical Science and Education, Karolinska Institute, Södersjukhuset, Stockholm, Sweden; 12 Department of Clinical Physiology, Clinical Sciences, Lund University, Skane University Hospital, Lund, Sweden

**Keywords:** acute myocardial infarction, epidemiology

## Abstract

**Objective:**

Most reports on the declining incidence of myocardial infarction (MI) during the COVID-19 have either been anecdotal, survey results or geographically limited to areas with lockdowns. We examined the incidence of MI during the COVID-19 pandemic in Sweden, which has remained an open society with a different public health approach fighting COVID-19.

**Methods:**

We assessed the incidence rate (IR) as well as the incidence rate ratios (IRRs) of all MI referred for coronary angiography in Sweden using the nationwide Swedish Coronary Angiography and Angioplasty Registry (SCAAR), during the COVID-19 pandemic in Sweden (1 March 2020–7 May 2020) in relation to the same days 2015–2019.

**Results:**

A total of 2443 MIs were referred for coronary angiography during the COVID-19 pandemic resulting in an IR 36 MIs/day (204 MIs/100 000 per year) compared with 15 213 MIs during the reference period with an IR of 45 MIs/day (254 MIs/100 000 per year) resulting in IRR of 0.80, 95% CI (0.74 to 0.86), p<0.001. Results were consistent in all investigated patient subgroups, indicating no change in patient category seeking cardiac care. Kaplan-Meier event rates for 7-day case fatality were 439 (2.3%) compared with 37 (2.9%) (HR: 0.81, 95% CI (0.58 to 1.13), p=0.21). Time to percutaneous coronary intervention (PCI) was shorter during the pandemic and PCI was equally performed, indicating no change in quality of care during the pandemic.

**Conclusion:**

The COVID-19 pandemic has significantly reduced the incidence of MI referred for invasive treatment strategy. No differences in overall short-term case fatality or quality of care indicators were observed.

## Introduction

COVID-19 has emerged as a global pandemic and public health crisis worldwide. Sweden’s first case of COVID-19 was reported on 4 February 2020 and by 1 March 2020, only 14 patients had acquired the disease. On 10 March 2020, community transmission was considered apparent and by 16 March 2020, the Swedish Public Health Agency (PHA) recommended all septuagenarians and older to stay at home. Since then more than 90 000 individuals have contracted disease in Sweden, resulting in over 5000 deaths.^1^ Efforts are currently made to better understand the cardiovascular effects of COVID-19.^2 3^ Recent reports indicate that fewer patients present to hospitals with myocardial infarction (MI) while the incidence of cardiac arrest have increased in hard-hit areas, raising the question if fear of acquiring COVID-19 results in healthcare avoidance with consequent higher cardiac-related mortality.^4–9^ However, most reports have either been anecdotal, survey results or geographically limited to areas with lockdowns as a mean of regressing disease transmission. We examined the incidence of MI during the ongoing COVID-19 pandemic in Sweden, which remained an open society with a different public health approach fighting COVID-19. Using the nationwide Swedish Coronary Angiography and Angioplasty Registry (SCAAR), we investigated how the COVID-19 pandemic influenced the incidence of patients presenting to hospitals with MI in relation to the same days 2015–2019.

## Methods

### Data sources and study cohort

The SCAAR register is part of the nationwide Swedish Web-system for Enhancement and Development of Evidence-based care in Heart Disease Evaluated According to Recommended Therapies (SWEDEHEART) registry.^10^ All coronary angiographies with subsequent interventions in the 29 catheterisation laboratories in Sweden are recorded in the SCAAR database for complete coverage of angiographies and percutaneous coronary interventions (PCIs) in Sweden. High granularity data on baseline demographics, comorbidities, indications, periprocedural medication as well as data pertaining to each coronary artery segment and various aspects of coronary intervention are collected by an interventional cardiologist answering mandatory questions. Data capture is web-based and allows for fast extraction of data to assure instant assessment of quality of care. Using SCAAR, we included all MIs referred for coronary angiography (henceforth referred to as MI) that occurred between 1 March and 7 May 2020 and MIs on the same days of the years 2015–2019 as reference. Data on death were obtained from the National Population Registry with data available up until 16 April 2020. COVID-19 reports were obtained from the Swedish PHA and are available online from the PHA website (www.folkhalsomyndigheten.se).

### Study design

We assessed the incidence rates for MI interventions between 1 March and 7 May 2020 (COVID-19 pandemic, 68 days) and those that occurred during 1 March–7 May during the consecutive years 2015–2019 (reference period), consisting of 340 days. The incidence rate ratio (IRR) between the two time periods was calculated. Separate analyses on the COVID-19 hotspot area in Sweden, Stockholm. This study adheres to the STROBE (Strengthening the Reporting of Observational Studies in Epidemiology) guidelines for observational studies.

### Outcomes

We assessed MI interventions as the primary outcome with interventions relating to ST-elevation myocardial infarction (STEMI) and non-ST-elevation myocardial infarction (NSTEMI) as secondary measures. The diagnosis of MI was set by interventional cardiologists according to the fourth universal definition of MI.^11^ All-cause mortality within 7 days was assessed as the primary outcome measure.

### Statistical analysis

Continuous variables are presented as medians and IQR and differences between groups were assessed with the Mann-Whitney U test. Categorical variables are displayed as counts and percentages and differences between groups were assessed using the χ^2^ test. A Poisson regression model was fitted as the primary statistical model to assess IRRs between the two time periods. Zero inflation was assessed visually using histograms and did not restrain the models. All models were tested for goodness-of-fit using the deviance goodness-of-fit and Pearson goodness-of-fit. When overdispersions was present, a negative binomial regression model was used instead. Incidence rates are reported as daily incidence rates of MI (absolute numbers) and incidence rate per 100 000 inhabitants per year. A calculation example of how incidence rates per 100 000 inhabitant per year were calculated can be found in the supplementary materials together with a table with number of patients at risk (online supplemental table 1). Results from regression analyses are reported as IRR with 95% CI and interpreted as difference in incidence in MI this year compared with the reference period. A sensitivity analyses was done adjusting for day of week as a categorical variable. Mortality was assessed using mortality event rates calculated with Kaplan-Meier estimates and HRs with 95% CIs were calculated using Cox proportional regression. An adjusted Cox proportional regression model was fitted, adjusting for differences in baseline demographics.

10.1136/heartjnl-2020-317685.supp1 Supplementary data

All statistical analyses were performed using Stata V.16.1 for Macintosh (Stata, Texas, USA). A two-sided p-value <0.05 was considered statistically significant.

### Subgroup analysis

The incidence of MI interventions was investigated in prespecified subgroups. Subgroups were based on sex, age, the presence of risk factors such as diabetes, hypertension, hyperlipidaemia, history of MI and smoking status (active smoker vs past smoker/non-smoker).

## Results

### Study population

A total of 17 656 MIs were referred for angiography during the study period, corresponding to an incidence rate of 246 per 100 000 inhabitants per year. A total of 2443 of these occurred during the COVID-19 pandemic and 15 213 during the reference period 1 March–May 7 of the years 2015–2019. The baseline characteristics of the study populations is shown in table 1. The median age of the total study population was 70 years (IQR 61–77 years) and 32.6% were women. No differences in age or sex were noted among the patients with COVID-19 pandemic compared with the reference period patients, both nationwide as well as in the COVID-19 hotspot subgroups (table 1). Patients admitted during the pandemic had slightly lower rates of history of MI and coronary artery bypass grafting (CABG). The proportion of remaining comorbidities was similar between patients with disease onset during the COVID-19 pandemic and patients with onset during the reference period. The proportion of STEMI versus NSTEMI did not differ during the pandemic compared with the reference period in any of the populations (table 1). Significant angiographic differences were noted on a nationwide level with lower rates of multivessel disease, during the pandemic compared with the reference period (table 1). Significantly fewer patients were referred to CABG during the COVID-19 pandemic (table 1).

**Table 1:** Baseline characteristics

	Total	All counties			Stockholm only		
		Control period	Pandemic	P value	Control period	Pandemic	P value
N (%)	17 656	15 213 (86.2)	2443 (13.8)		2597 (87.0)	387 (13.0)	
Age (years), median (IQR)	70 (61–77)	70 (61–77)	70 (61–77)	0.25	68 (59–76)	68 (59–75)	0.36
Men, n (%)	11 894 (67.4)	10 248 (67.4)	1646 (67.4)	0.99	1805 (69.5)	274 (70.8)	0.60
Women, n (%)	5762 (32.6)	4965 (32.6)	797 (32.6)		792 (30.5)	113 (29.2)	
BMI, median (IQR)	26.8 (24.2–30.9)	26.8 (24.2–30.0)	26.9 (24.3–30.1)	0.08	26.8 (24.2–30.0)	26.9 (24.1–30.1)	0.98
Current smoker, n (%)	3376 (19.1)	2928 (19.3)	448 (18.3)	0.57	507 (19.5)	69 (17.8)	0.26
Medical history							
Diabetes, n (%)	3804 (21.6)	3277 (21.5)	527 (21.6)	0.09	594 (22.9)	81 (20.9)	0.46
Hypertension, n (%)	10 329 (59.1)	8967 (58.9)	1462 (59.8)	0.04	1529 (58.9)	221 (57.1)	0.2
Hyperlipidaemia, n (%)	6972 (39.5)	6043 (39.7)	929 (38.0)	0.03	990 (38.1)	131 (33.9)	0.26
MI, n (%)	3801 (21.5)	3322 (21.8)	479 (19.6)	≤0.001	583 (22.5)	77 (19.9)	0.15
PCI, n (%)	3099 (17.6)	2659 (17.5)	449 (18.0)	0.66	451 (17.4)	72 (18.6)	0.55
CABG, n (%)	1140 (6.5)	1014 (6.7)	126 (5.2)	0.002	167 (6.4)	15 (3.9)	0.005
In-hospital characteristics							
STEMI, n (%)	6713 (38.0)	5814 (38.2)	899 (36.8)	0.18	933 (35.9)	144 (37.2)	0.62
NSTEMI, n (%)	10 943 (62.0)	9399 (61.8)	1544 (63.2)	0.18	1664 (64.1)	243 (62.8)	0.62
STEMI after cardiac arrest, n (%)	310 (1.8)	270 (1.8)	40 (1.6)	0.63	53 (2.0)	7 (1.8)	0.76
Time from symptom to PCI (min), median (IQR)	930 (200–2461)	931 (200–2495)	820 (198–2130)	0.04	948 (180–2299)	797 (225–1822)	0.55
Time from first ECG to PCI (min), median (IQR)	650 (83–1890)	646 (83–1926)	582 (79–1599)	0.01	721 (77–1725)	806 (77–1527)	0.59
Duty hours, n (%)	10 806 (61.2)	9281 (61.0)	1525 (62.4)	0.11	1649 (63.5)	231 (59.7)	0.003
Killip class, n (%)							
1	14 221 (80.5)	12 234 (80.4)	1987 (81.3)	0.29	1895 (73.0)	293 (75.7)	0.83
2	562 (3.2)	489 (3.2)	73 (3.0)		84 (3.2)	11 (2.8)	
3	164 (0.9)	150 (1.0)	14 (0.6)		27 (1.0)	3 (0.8)	
4	256 (1.5)	218 (1.4)	38 (1.6)		45 (1.7)	7 (1.8)	
High-sensitivity troponin T (ng/L), median (IQR)	560 (155–2133)	574 (157–2144)	538 (146–2210)	0.85	578 (152–2289)	516 (126–2310)	0.70
Angiographic findings, n (%)							
Normal/atheromatosis	2622 (14.9)	2211 (14.5)	411 (16.8)	≤0.001	466 (17.9)	71 (18.4)	0.26
1VD not LM	6553 (37.1)	5611 (37.9)	942 (38.6)		990 (38.1)	165 (42.6)	
2VD not LM	4012 (22.7)	3444 (22.6)	568 (23.3)		521 (20.1)	79 (20.4)	
3VD not LM	3059 (17.3)	2710 (17.8)	349 (14.3)		417 (16.1)	46 (11.9)	
LM or LM including 1-3VD	1376 (7.8)	1210 (8.0)	166 (6.8)		199 (7.7)	25 (6.5)	
PCI, n (%)	11 696 (66.2)	10 049 (66.1)	1647 (67.4)	0.19	1651 (63.6)	261 (67.4)	0.14
Primary decision CABG, n (%)	1017 (5.8)	911 (6.0)	106 (4.3)	≤0.001	114 (4.4)	8 (2.1)	0.03

BMI, body mass index; CABG, coronary artery bypass grafting; LM, left main coronary artery; MI, myocardial infarction; NSTEMI, non-ST-elevation myocardial infarction; PCI, percutaneous coronary intervention; STEMI, ST-elevation myocardial infarction; VD, vessel disease.

### Incidence rates and ratios

**Figure 1:**
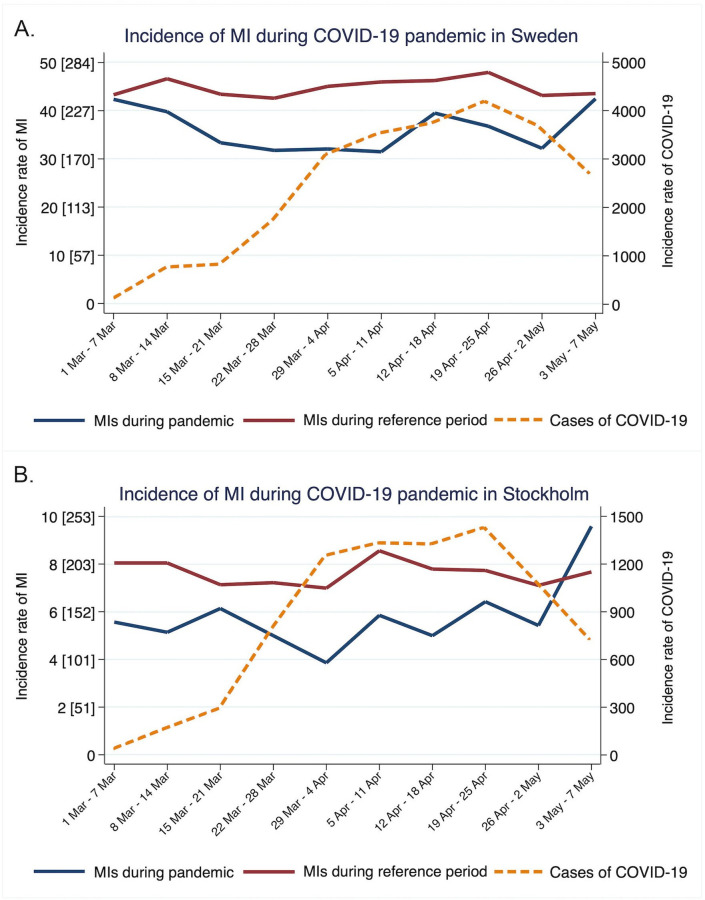
Incidence rate of myocardial infarction (MI) interventions and COVID-19 in Sweden as well as its capital city Stockholm. (A) Visualises the incidence rate of MI for each 7-day period during COVID-19 pandemic (1 March–May 2020) and the reference period (1 March 1–7 May, the years 2015–2019) together with the incidence of COVID-19 in Sweden. The incidence of MI is presented as daily incidence (absolute numbers) and the incidence rate per 100 000 inhabitants per year in brackets. (B) Visualised the same information but for Stockholm county. A clear decline in MI incidence can be observed since the beginning of the pandemic both nationwide and isolated to Stockholm. On 12 April, a national campaign was launched throughout major newspapers, television channels, on the web and social media, aimed to inform and encourage patients with symptoms suggestive of MI to seek medical care. The inflow of patients with MI returned to typical levels both nationally as well as in Stockholm by 7 May 2020 reflecting how adequate countermeasures can reverse the indirect effects of COVID-19 pandemic on healthcare-seeking behaviour.

**Figure 2:**
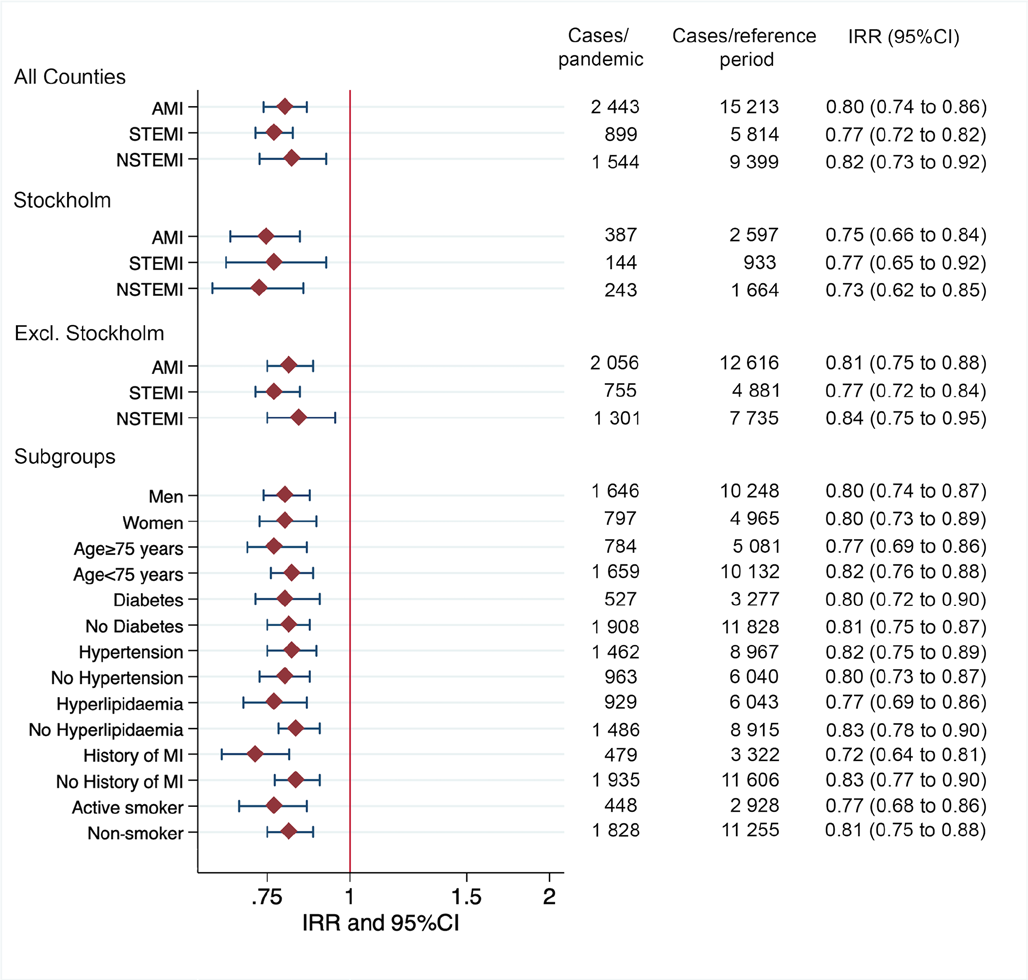
Results of primary, secondary and subgroup analyses. Forest plot showing the incidence rate ratios (IRRs) of myocardial infarction (MI) during COVID-19 pandemic in Sweden compared with the reference period together with absolute number during the given time period (COVID-19 pandemic vs reference period). The daily incidence rate in absolute numbers can be calculated by dividing events by days at risk (COVID-19 pandemic=68; reference period=340). A conversion formula and calculation example to convert daily incidence rates to incidence rate per 100 000 inhabitants can be found in the online supplemental material. AMI, acute myocardial infarction; NSTEMI, non-ST-elevation myocardial infarction; STEMI, ST-elevation myocardial infarction.

Figure 1 shows the incidence of all MI interventions as well as stratified by STEMI and NSTEMI during the COVID-19 pandemic in relation to the same dates 2015–2019 together with the incidence rate of COVID-19 in Sweden. Figure 2 shows the IRR for all study populations as well as stratified by patient subgroups. The daily incidence rate of MI interventions during the COVID-19 pandemic was 36 MIs per day, translating into 204 MIs per 100 000 per year. The daily incidence rate of MI interventions during the reference period was 45 MIs per day, translating into 254 MIs per 100 000 per year, resulting in IRR=0.80, 95% CI (0.74 to 0.86), p<0.001 (figure 2). The results for STEMI interventions were 13 vs 17 per day (IRR=0.77, 95% CI (0.72 to 0.82), p<0.001) and for NSTEMI 23 vs 28 per day (IRR=0.82, 95% CI (0.73 to 0.92), p=0.003) (figure 2). For Stockholm county, the incidence rate reductions for total MI interventions were (IRR=0.75, 95% CI (0.66 to 0.84), p<0.001) more pronounced for STEMI (IRR=0.77, 95% CI (0.65 to 0.92), p<0.001) compared with NSTEMI (IRR=0.73, 95% CI (0.62 to 0.85), p<0.001). Similar results were obtained in the expanded COVID-19 hotspot area (online supplemental table 2). After excluding COVID-19 hotspot areas, there remained a nationwide significant reduction in MI intervention incidence during the pandemic (figure 2 and online supplemental table 2). Results were consistent across subgroup analyses based on sex, age, as well as the presence of risk factors such as diabetes, hypertension, history of MI and smoking as well as after adjusting for day of week (figure 2 and online supplemental table 3).

### Case fatality

**Figure 3:**
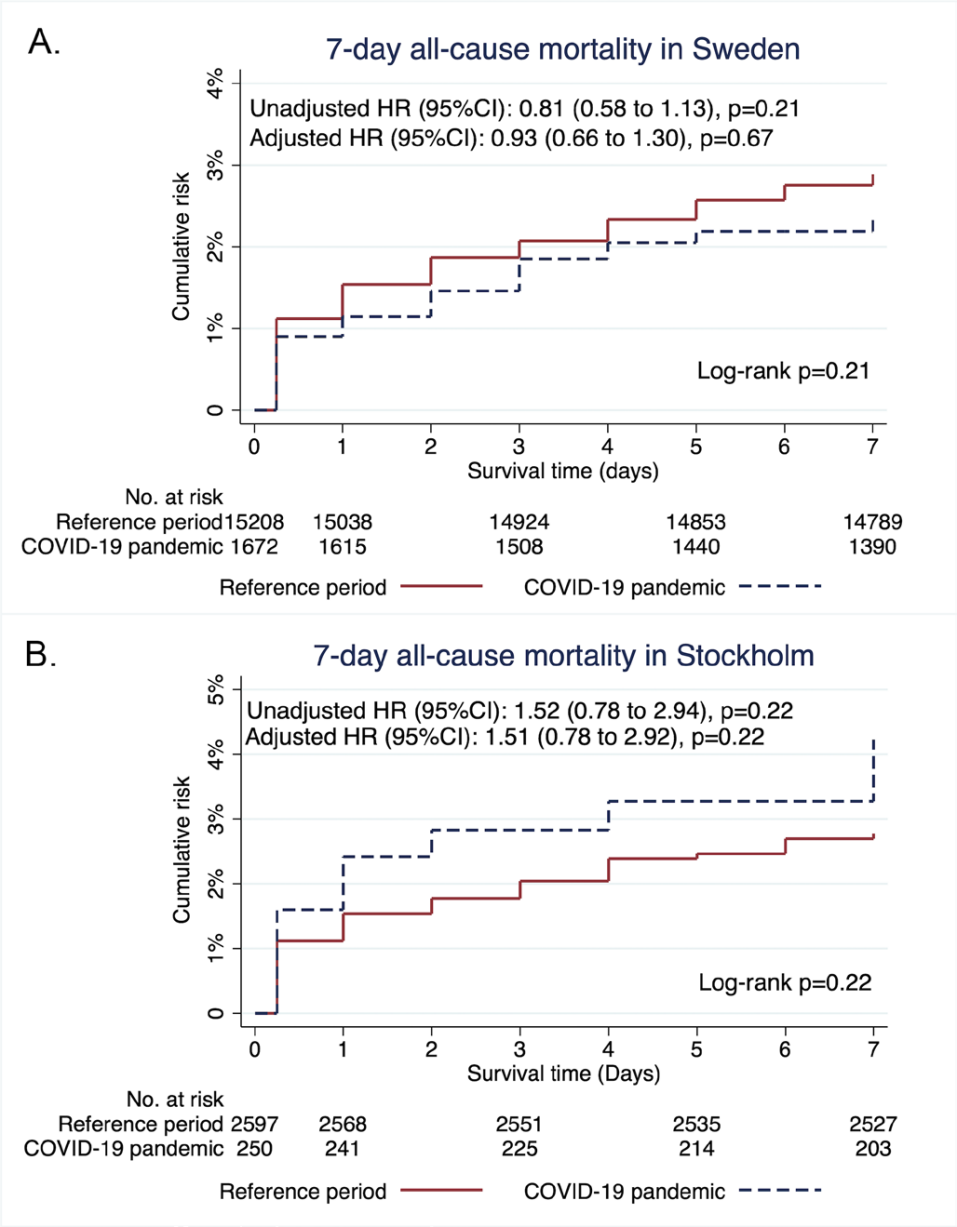
1-survival graph showing case fatality within 7 days during the pandemic compared with the reference period. (A) shows the failure estimates for the entire population stratified by pandemic and reference period together with results from Cox proportional regression models. No difference in case fatality can be observed. (B) shows the failure estimates for Stockholm county stratified by pandemic and reference period together with results from Cox proportional regression models. The adjusted models were adjusted for baseline differences found in table 1 except for time from symptom onset and first ECG to percutaneous coronaryintervention.

No difference was observed in Kaplan-Meier event rates of all cause-mortality within 7 days in the entire country during COVID-19 pandemic as compared with during the reference period 439 (2.3%) compared with 37 (2.9%) (unadjusted HR: 0.81, 95% CI=0.58 to 1.13), p=0.21 (figure 3A). Results were consistent after adjusting for confounders (history of MI, previous CABG and angiographic coronary findings) (adjusted HR: 0.93, 95% CI=0.66 to 1.30), p=0.67, figure 3A. A trend toward higher mortality can be seen in Stockholm, with Kaplan-Meier event rates 10 (4.2%) compared with 72 (2.8%) (unadjusted HR: 1.52, 95% CI=0.78 to 2.94), p=0.22 (figure 3B). Results were consistent after adjusting for confounders observed in baseline differences in Stockholm (adjusted HR: 1.51, 95% CI=0.78 to 2.92), p=0.22 (figure 3B). Subgroup analyses of case fatality of STEMI and NSTEMI independently are presented in online supplemental table 4, showing consistent results except for STEMI in Stockholm. Case fatality within 7 days after STEMI was significantly higher in Stockholm during the pandemic compared with the reference period 10 (12.3%) compared with 5 (5.9%) (unadjusted HR: 2.08, 95% CI=1.06 to 4.09), p=0.03 (online supplemental table 4).

### Quality of coronary care during COVID-19 pandemic

Significant differences were observed in time from first medical contact or time from symptom onset to coronary angiography (table 1). Time from symptom onset to PCI was shorter during COVID-19 pandemic (820 min (IQR: 198–2130) compared with 921 min (IQR: 200–2495), p=0.04) (table 1). A shorter time from first ECG to PCI was observed as well (582 min (IQR: 79–1599) compared with 646 min (IQR: 83–1926), p=0.01). On stratifying MI interventions into STEMI, no difference was observed in time from symptom onset to PCI but shorter time from first ECG to PCI was observed during the pandemic (70 min (IQR: 48–105) compared with 75 min (IQR: 53–119), p=0.02) (table 2). Both time variables were shorter for NSTEMI during COVID-19 pandemic compared with the reference period (table 2). Rates of PCI were numerically higher although not statistically significant during COVID-19 pandemic compared with reference period (table 1). No difference in proportions of PCI performed or cardiac enzymes were observed on stratifying MI interventions into STEMI and NSTEMI. No difference in any time variable, proportions of PCI performed or cardiac enzymes were observed after stratifying MI interventions into STEMI and NSTEMI in Stockholm county.

**Table 2:** Quality of care indicators stratified on STEMI and NSTEMI

Quality of care indicators	STEMI			NSTEMI		
	Reference period	Pandemic	P value	Reference period	Pandemic	P value
Sweden						
Time from symtom to PCI (min), median (IQR)	190 (122–385)	191 (116–401)	0.45	2070 (1260–3780)	1825 (1069–2986)	<0.001
Time from first ECG to PCI (min), median (IQR)	75 (53–119)	70 (48–105)	0.02	1600 (959–2986)	1422 (855–2430)	<0.001
High-sensitivity troponin T (ng/L), median (IQR)	1955 (596–4853)	2186 (516–5407)	0.23	282 (109–799)	271 (99–730)	0.57
PCI, n (%)	4788 (82.4)	769 (85.5)	0.019	5261 (56.0)	878 (56.9)	0.51
Stockhholm						
Quality of care indicators						
Time from symptom to PCI (min), median (IQR)	180 (110–390)	180 (112–343)	0.94	1968 (1259–3570)	1570 (1057–2980)	0.08
Time from first ECG contact to PCI (min), median (IQR)	64 (45–115)	57 (41–95)	0.13	1504 (930–2819)	1377 (878–2263)	0.06
High-sensitivity troponin T (ng/L), median (IQR)	2485 (893–5235)	2690 (766–7340)	0.28	229 (90–657)	179 (72–671)	0.30
PCI, n (%)	754 (80.8)	120 (83.3)	0.47	897 (53.9)	141 (58.0)	0.23

NSTEMI, non-ST-elevation myocardial infarction; PCI, percutaneous coronary intervention; STEMI, ST-elevation myocardial infarction.

## Discussion

We investigated the incidence and outcome of MI during the current COVID-19 pandemic in a real-time setting in Sweden. Our results showed a nationwide and significant reduction in the incidence of MIs referred for an invasive treatment strategy since the outbreak of COVID-19. No difference in case fatality or quality indicators were observed during the pandemic compared with reference period. Similar results were observed in Sweden’s COVID-19 hotspot area Stockholm and other areas.

External triggers such as during natural disasters, wars, sports events, stock market crashes, cold weather, national holidays or influenza pandemics have repeatedly been associated with a short-term peak in MI incidence.^12–18^ However, in contrast to these external triggers of MI, the current pandemic has resulted in declining rates of MI. To date three reports and two studies has been published on this topic, reporting reductions in MI admissions.^4 6–9^ The most detailed studies include a multicentre survey study from Italy and a nationwide analysis of acute coronary syndrome (ACS) admissions in England. Important differences in study design as well as findings warrant highlighting.^8 9^ The survey study by De Rosa *et al* included patients admitted during a 1-week period of the pandemic compared with equivalent week in 2019 whereas the nationwide analysis of ACS admissions by Mafham *et al* used International Classification of Diseases codes. Both studies report overall reductions of similar proportion as we observed in Sweden but with a significantly higher reduction in NSTEMI (Italy: 65% and England: 42%). In addition, De Rosa *et al* also assessed case fatality which was significantly higher during the pandemic, both overall and isolated to patients with STEMI.^9^ Overall case fatality was not increased during the pandemic in Sweden nor in Stockholm, but alarmingly the case fatality of STEMI in Stockholm was twice as high and in line with findings from Italy.^9^ These differences could be attributed to study design or study period as well as relate to the countries being in a different stage in the pandemic. Italy had an earlier outbreak as well as a more severe outbreak compared with Sweden. Sweden is one of few countries that have not had a lockdown and it cannot be ruled out that a more open society may have resulted in a different outcome of the indirect consequences of the pandemic.

The reasons behind the declining rates of MI are probably multifactorial. Fear of acquiring COVID-19 could result in fewer patients with MI seeking acute cardiac care. In addition, patients misinterpreting MI symptoms such as dyspnoea as a symptom of COVID-19 might avoid seeking healthcare in favour of self-quarantine. We observed similar results across subgroups based on sex, age, as well as the presence of risk factors such as diabetes, hypertension, history of MI, and smoking, indicating no change in patient category seeking cardiac care. Vigorous social isolation and improved hand hygiene might minimise the presence of short-term risk factors in high-risk patients. For example, accumulating evidence suggests influenza as a strong trigger of MI, with reports of up to sixfold short-term increase in MI risk.^19 20^ With the emergence of the COVID-19 pandemic, the incidence of other viruses causing respiratory tract infections have significantly decreased, probably as a result of social distancing and improved hand hygiene and possibly contributing to the decline in MI incidence.^21^ Furthermore, self-isolation, working from home and more gentle recreational habits could decrease stress and exertional-induced MIs in individuals at risk. The decline in MI interventions were equally pronounced in the COVID-19 hotspot area of Stockholm, accounting for more than 40% of all COVID-19 cases in Sweden. Results were consistent after excluding Stockholm as well as the expanded hotspot areas from the analyses reflecting an overall nationwide decline in MI incidence, even in areas with low incidence of COVID-19. In our opinion, these results in indirect consequences of the pandemic rather than a direct disease-related effect.

Could changes in hospital strategies explain the reduction of MI referred for an invasive treatment strategy? So far, no national strategy changes have been made after the outbreak of COVID-19. Furthermore, the MI reduction was pronounced for STEMI as well where the indication for angiography is firmer. Both patient and medical delays were shorter during the pandemic and similar rates of PCI were performed during the pandemic, reflecting continued medical vigilance on patients seeking cardiac care. We performed a separate analysis outside the frames of the present study assessing referral to angiography among patients admitted to a Coronary care unit (CCU) using the SWEDEHEART CCU registry. Results of this analysis should be interpreted cautiously due to report delay to the registry. However, this analysis showed a small increase in coronary angiogram referrals in patients discharged with MI diagnosis during the pandemic compared with reference period (85% compared with 82.5%, p=0.015). Similarly, proportions of patients undergoing angiography within 0–1 days from admission to CCU were higher during the pandemic 70.4% compared with 63.7%, p<0.001. Therefore, we find it unlikely that a more restrictive invasive approach explains our results. However, fewer patients were referred to CABG during the pandemic (table 1). If this could be explained by fewer patients with multivessel disease, seeking healthcare or indicative of a scarcity of intensive care beds is unknown.

The objective of the SWEDEHEART registry is to monitor quality of cardiac care. By Easter holiday we observed that the incidence rate of MI was down by 21% in Sweden and 34% in Stockholm. More alarmingly, the incidence of STEMI in Stockholm was reduced by 40% at this time. This information prompted a national campaign coordinated by the Swedish Society of Medicine, the Swedish Society of Cardiology, the Swedish Heart and Lung Foundation and the SWEDEHEART registry on 12 April 2020. The campaign was launched in all major newspapers, television channels in Sweden, on the web, as well as social media and aimed to raise awareness of MI symptoms and encourage patients to seek medical care. Four weeks after the campaign was initiated, the inflow of patients with MI returned to typical levels with an increased MI incidence from 35 to 42 MIs per day in the country. During the same time, COVID-19 cases continued to increase. These results were observed both nationally as well as in Stockholm (figure 1B). We believe that information campaigns using several media platforms, and coordinated by authorities and healthcare providers could help to reverse indirect harmful effects of the COVID-19 pandemic on healthcare-seeking behaviour.

Our study has a number of notable strengths but also limitations. It includes all MIs referred for a coronary angiography in Sweden from a validated nationwide register including patients in an all-comer setting with diagnosis of MI and its subtypes set by an experienced cardiologist. The decline in MI interventions raises the question of whether more patients have been conservatively treated. Although this cannot be completely ruled out given the nature of this study, we did not find evidence of any change in invasive treatment strategies or any change in quality of care during the pandemic. Finally, it is difficult to estimate the effect of competing risk with COVID-19. However, the effect of MI reduction began early in the pandemic when infection and COVID-19 fatality rates were low.

In conclusion, the COVID-19 pandemic has had a significant impact on the incidence of MI referred for invasive treatment strategy in Sweden. No differences in overall case fatality or quality indicators were observed. These findings reflect the importance of continuous monitoring of both the direct and indirect effects of the COVID-19 pandemic to allow expedited countermeasures.

**Key messages**

**What is already known on this subject?**

- There has been reports of declining incidence of myocardial infarction (MI) and increased fatality rates. However, most reports have either been anecdotal, survey results or geographically limited to areas with lockdowns.

**What might this study add?**

- The present study confirms a stable decline in incidence of MI during the COVID-19 pandemic in Sweden, which has taken a different public health approach to fight COVID-19 by remaining an open society. Our results show no difference in overall short-term case fatality or quality of care indicators, indicating that patients are being treated equally effective as prior to the pandemic. This study provides important information on vital functions of the cardiac care in Sweden.

**How might this impact on clinical practice?**

- Patients not seeking acute cardiac care due to MI may suffer from larger infarcts, malignant arrhythmias and severe heart failure and cause a major healthcare challenge. This calls for continuous monitoring of the direct and indirect effects of the COVID-19 pandemic to raise healthcare awareness and allow for expedited countermeasures to reverse incidental harmful effects of the pandemic.
