# Supplementary material for: Incidence and outcome of myocardial infarction treated with percutaneous coronary intervention during COVID-19 pandemic
Source: Heart. 2020 Oct 6;106(23):1812–8. doi: 10.1136/heartjnl-2020-317685 (PMC7677488; doi:10.1136/heartjnl-2020-317685)
Supplement: Supplementary data [file heartjnl-2020-317685supp001.pdf]

Incidence of myocardial infarction during COVID-19 pandemic in Sweden

Supplementary Files

Incidence calculation formula 2
Supplementary Table 1.. 3
Supplementary Table 2.. 4
Supplementary Table 3.. 5
Supplementary Table 4.. 6

Incidence calculation formula

The average population size aged ≥ 30 years in Sweden during study period which is 6 432 704 according to Statistics Sweden http://www.scb.se/en/), the nationwide statistics agency, see Supplementary Table 2.

1) Incidence rate of MI during Covid-19 pandemic:

Incidence rate= Number of new events/time at risk

New MIs during Covid-19 pandemic=1 364.
Days at risk (March 1 - April 8, 2020)=39.
1 364/39=34.97MIs per day.

Patients at risk=6 432 704.
MI per 100 000 inhabitants per day during Covid-19 pandemic
= (34.97/6432704)*100000=0.54

This translates into 0.54*365=197 MIs per 100 000 inhabitants per year.

2) Similar calculation for the reference period:

New MIs during the reference period=8676
Days at risk (March 1 - April 8, consecutive years 2015-2019)=195
8676/195=44.49 MIs per day.

Patients at risk=6 432 704.
MI per 100 000 inhabitants per day during Covid-19 pandemic
= (44.49 /6432704)*100000=0.69

This translates into 0.69*365=252 MIs per 100 000 inhabitants per year.

Supplementary Table 1.

Table showing number of patients at risk for each county and in total. Patients at risk are individuals ≥30 years. Numbers are based on average population size during the study period.

County	Patients at risk
Stockholm	1440268
Uppsala	225630
Sodermanland	188135
Ostergotland	286894
Jonkoping	224784
Kronoberg	124240
Kalmar	161247
Gotland	39752
Blekinge	104011
Skane	849756
Halland	209415
Vastra Gotaland	1069766
Varmland	184821
Orebro	188921
Vastmanland	174397
Dalarna	188919
Gavleborg	188242
Vasternorrland	163136
Jamtland	85086
Vasterbotten	168937
Norrbotten	166347
Total:	6432704

Supplementary Table 2.

Results for expanded Covid-19 hotspot area including the three most Covid-19 afflicted hotspot counties (Stockholm, Södermanland and Östergötland).

Supplementary Table 2.	Cases/ pandemic	Cases/reference period	IRR	LCL	UCL
Expanded hotspot					
MI	3881	546	0.70	0.63	0.78
STEMI	1471	218	0.74	0.64	0.85
NSTEMI	2410	328	0.68	0.59	0.79
Excl. expanded hotspot					
MI	11332	1897	0.84	0.77	0.91
STEMI	4343	681	0.78	0.72	0.85
NSTEMI	6989	1216	0.87	0.77	0.98

Supplementary Table 3.

Results of analyses adjusted for day of week as a categorical variable.

Supplementary Table 3.	Cases/ pandemic	Cases/reference period	IRR	LCL	UCL
Sweden					
MI	15213	2443	0.80	0.76	0.84
STEMI	5814	899	0.77	0.72	0.83
NSTEMI	9399	1544	0.82	0.77	0.88
Stockholm					
MI	2597	387	0.74	0.67	0.83
STEMI	933	144	0.77	0.65	0.92
NSTEMI	1664	243	0.73	0.63	0.83

Supplementary Table 4.

Results of case fatality within 7 days due any cause during Covid-19 pandemic and reference period for all MI interventions as well as for STEMI and NSTEMI independently.

Supplementary table 4.

**Sweden**	Reference period	Pandemic	Log rank	Unadjusted HR (95%CI)	Adjusted* HR (95%CI)
Case fatality within 7 days					
MI	439 (2.9%)	37 (2.3%)	0.2	0.81 (0.58-1.13), p=0.21	0.93 (0.66-1.31), p0.68
STEMI	334 (5.8%)	29 (4.9%)	0.39	0.85 (0.58-1.24), p=0.40	1.00 (0.68-1.48), p=0.99
NSTEMI	105 (1.1%)	8 (0.8%)	0.39	0.73 (0.36-1.50), p=0.39	0.80 (0.39-1.64), p=0.54

*adjusted for history of MI, previous CABG and coronary anatomy findings on angiogram

**Stockholm**	Reference period	Pandemic	Log rank	Unadjusted HR (95%CI)	Adjusted** HR (95%CI)
Case fatality within 7 days					
MI	72 (2.8%)	10 (4.2%)	0.21	1.52 (0.78-2.94), p=0.22	1.51 (0.78-2.92), p=0.22
STEMI	55 (5.9%)	10 (12.3%)	0.03	2.08 (1.06-4.09), p=0.03	2.07 (1.05-4.06), p=0.035
NSTEMI	17 (1.0%)	0 (0%)	0.21	NA	NA

**adjusted for previous CABG and angiography during duty hours
